# Signalling Pathways Implicated in Alzheimer′s Disease Neurodegeneration in Individuals with and without Down Syndrome

**DOI:** 10.3390/ijms21186906

**Published:** 2020-09-20

**Authors:** Carmen Martínez-Cué, Noemí Rueda

**Affiliations:** Department of Physiology and Pharmacology, Faculty of Medicine, University of Cantabria, 39011 Santander, Spain; ruedan@unican.es

**Keywords:** Down syndrome, Alzheimer’s disease, signalling pathways, neurodegeneration

## Abstract

Down syndrome (DS), the most common cause of intellectual disability of genetic origin, is characterized by alterations in central nervous system morphology and function that appear from early prenatal stages. However, by the fourth decade of life, all individuals with DS develop neuropathology identical to that found in sporadic Alzheimer’s disease (AD), including the development of amyloid plaques and neurofibrillary tangles due to hyperphosphorylation of tau protein, loss of neurons and synapses, reduced neurogenesis, enhanced oxidative stress, and mitochondrial dysfunction and neuroinflammation. It has been proposed that DS could be a useful model for studying the etiopathology of AD and to search for therapeutic targets. There is increasing evidence that the neuropathological events associated with AD are interrelated and that many of them not only are implicated in the onset of this pathology but are also a consequence of other alterations. Thus, a feedback mechanism exists between them. In this review, we summarize the signalling pathways implicated in each of the main neuropathological aspects of AD in individuals with and without DS as well as the interrelation of these pathways.

## 1. Introduction

Alzheimer’s disease (AD), the most common form of dementia, affects 44 million people worldwide [1]. The progressive loss of cognitive abilities in this condition is associated with neuropathological changes, including the accumulation of amyloid plaques comprising β-amyloid (Aβ) peptides and neurofibrillary tangles (NFTs) formed by insoluble deposits of abnormally hyperphosphorylated tau, and synapse and neuron loss.

Down syndrome (DS), caused by a partial or complete triplication of human chromosome 21 (Hsa21), affects more than 6 million persons globally [2]. The cognitive alterations found in DS are primarily caused by prenatal changes in central nervous system (CNS) growth and differentiation [3,4]. However, by the fourth decade of their lives, all individuals with DS develop AD neuropathology identical to that found in individuals with sporadic AD [3,5,6,7,8,9]. In the DS population, AD is likely to arise due to the genetic imbalance of several trisomic genes and the interplay of these triplicated genes with other diploid genes. However, in sporadic AD, the main genetic risk factor is the presence of the E4 allele of apolipoprotein E (ApoE) [10]. Despite the different genetic etiopathology of AD in both conditions, the aforementioned fact that all DS individuals develop AD neuropathology suggests that common downstream signalling pathways are affected in both disorders. Thus, the study of the mechanisms implicated in the early onset and high prevalence of AD in DS could be extremely useful in understanding the etiopathology of neurodegeneration and related dementia in sporadic AD.

Some pathological events that appear years before the appearance of amyloid plaques and NFTs play an important role in the onset of the main neuropathological characteristics of AD. These alterations include neuroinflammation, cellular senescence, altered proteostasis, oxidative stress, and reduced neurogenesis [11,12,13,14,15]. Numerous studies have demonstrated the role of these early alterations in the increase in Aβ burden, tau hyperphosphorylation, neuronal death, and accelerated cognitive decline [16,17,18,19,20,21,22,23]. Several signalling pathways are implicated in the onset and aggravation of the aforementioned pathological changes characteristic of AD in individuals with or without DS. This review summarizes the implication of these pathways and their interplay on the most relevant aspects of this disease, including amyloid plaques, NFTs, cholinergic degeneration, oxidative stress, mitochondrial dysfunction, disturbed energy metabolism, cellular senescence, neuroinflammation, altered neurogenesis, and impaired neurotransmission.

Also, some signalling pathways play a role in numerous neuropathological aspects of AD. Because several feedback loops exist between them, their interplay aggravates AD pathology. Thus, we have emphasized the alterations of their function in different aspects of AD as well as their interactions, especially in amyloid plaque and NFT formation, oxidative stress, energy metabolism, neuroinflammation, neurotransmitter release, and synaptic dysfunction. Among these pathways are those controlled by the Dual Specificity Tyrosine-Regulated Protein Kinase 1 (DYRK1A), the Regulator of Calcineurin (RCAN1), neurotrophins, and the Mammalian Target of Rapamycin (mTOR). Finally, this review also describes the role of other pathways that are altered in specific AD signs (e.g., Superoxide Dismutase (SOD1) in oxidative stress or insulin signalling, glucose transport, and metabolism in altered energy metabolism, among others).

The purpose of this review is to provide an overview of the role of the most relevant signalling pathways implicated in the onset and progression of AD in individuals with and without DS.

## 2. Amyloid Plaques

AD is characterized by altered proteostasis since many of its pathological characteristics are due to changes in the balance and function of different proteins and peptides [15]. In particular, the accumulation of Aβ in plaques is produced by alterations in the synthesis, folding, and clearance of these peptides. 

In AD brains, one of the causes of the accumulation of Aβ aggregates is their defective clearance from the brain, a process normally facilitated by ApoE. Indeed, the major genetic risk factor for sporadic AD is a polymorphism of ApoE [10,24,25]. ApoE contributes to the maintenance of brain homeostasis through numerous pathways, including the regulation of cholesterol, glucose metabolism, synaptic plasticity, neurogenesis, inflammatory responses, and Aβ metabolism [26,27,28]. In the AD population, the presence of the ApoE4 isoform correlates with a higher probability of developing dementia and an earlier onset of cognitive decline [26]. 

The APOE genotype has also been found to modulate the age of onset and progression of AD in DS. DS carriers of the E4 allele have a greater risk of developing AD and an earlier onset of the disease when compared to carriers of other alleles [25,29].

Several Hsa21 genes are implicated in the altered proteostasis that leads to the changes in Aβ aggregation and clearance in AD. Aβ oligomers are the proteolytic products of the Amyloid Precursor Protein (APP) [30]. Because the gene that encodes APP maps to Hsa21, its overexpression was proposed to be responsible for the accumulation of Aβ in AD in individuals with and without DS [31]. However, compelling evidence demonstrates that other Hsa21 genes are key players in the development of AD neuropathology. Some of them encode kinases and phosphatases with multiple targets in different signalling pathways. 

One of the genes that has received increased attention is DYRK1A, which encodes a serine-threonine protein kinase [32] and has been associated with the cognitive impairment found in DS [33,34,35,36]. This gene plays a role in the amyloid pathology found in AD and DS. Individuals with AD display enhanced levels of DYRK1A mRNA [37]. DYRK1A phosphorylates APP and enhances its cleavage by β- and γ-secretases [38]. Also, DYRK1A phosphorylates presenilin (PS), the catalytic subunit of the γ-secretase complex [39]. Both phosphorylations promote APP-processing by the amyloidogenic pathway, increasing the formation of the peptides Aβ40 and Aβ42. In turn, these peptides increase DYRK1A transcription, leading to high levels of expression of this kinase in sporadic AD [40]. 

Another Hsa21-encoded gene that has been implicated in amyloid plaque accumulation is RCAN1, which encodes a calcium-activated serine/threonine protein phosphatase [41]. RCAN1 mediates Aβ-induced neuronal death by enhancing oxidative stress and by disrupting cellular calcium homeostasis in the AD brain [42]. RCAN1 expression is regulated by the calcineurin-Nuclear Factor of Activated T cells (NFAT) transcription factor signalling pathway [43]. In turn, RCAN1 overexpression inhibits different signalling pathways that are controlled by NFAT [44,45]. Thus, the chronic overexpression of RCAN1 that occurs in DS and AD [46] inhibits calcineurin and dysregulates the NFAT pathways. Lower levels of calcineurin and hyperphosphorylation of NFAT are found in the brains of these individuals [42,45,47,48]. The alterations in NFAT signalling promotes Aβ production through different mechanisms, including modulation of the expression of the β-site APP cleaving enzyme 1 (BACE1) gene implicated in Aβ production [49].

In addition, RCAN1 and DYRK1A act synergistically to control the phosphorylation of cytoplasmatic NFAT (NFATc). NFATc may be phosphorylated by DYRK1A, decreasing gene transcription activity [42,48].

Another kinase that has a central role in AD neuropathology is mTOR, a serine/threonine protein kinase. mTOR is involved in the regulation of the proteostasis network due to its ability to inhibit autophagy, a specialized degradative system for the removal of aggregated proteins [50]. In physiological conditions, mTOR inhibits the accumulation of toxic protein aggregates such as Aβ [51]. However, a role for altered mTOR signalling in amyloid pathology has been proposed [52]. mTOR is regulated and interacts with 5′AMP activated protein kinase (AMPK), Phosphoinositide 3 Kinase (PI3K)/AKT, glycogen synthase kinase (GSK3), the extracellular signal-regulated protein kinases (ERK1/2), and insulin/insulin growth factor (IGF) [51,53,54]. The PI3K/Akt/mTOR axis is hyperactivated in DS and AD [55,56,57,58,59,60,61,62,63] and contributes to the altered Aβ generation, deposition, and clearance found in these conditions [51,64,65,66,67,68,69,70,71,72]. In turn, Aβ activates the PI3K/Akt/mTOR signalling pathway [62,67,73,74,75], generating a feedback loop that further aggravates the amyloid pathology in individuals with AD with or without DS.

Another signalling pathway that has been implicated in AD neuropathology is the transcription factor cAMP response element-binding protein (CREB) [76]. CREB is phosphorylated and activated by PI3K/AKT, protein kinase A (PKA), and protein kinase C (PKC) [77]. However, GSK3β inactivates CREB [78,79], and since Aβ peptides activate GSK3β, their overproduction in AD reduces CREB activity [80,81,82]. In agreement with these data, patients with AD show decreased CREB phosphorylation due to alterations in cAMP/PKA signaling [82,83,84]. The disturbances in cAMP/PKA-dependent CREB signalling have been demonstrated to be responsible for Aβ-induced synaptic loss and cognitive impairments [82,85,86].

CREB also regulates several neurotrophins that play a crucial role in cognition such as Brain-Derived Neurotrophic Factor (BDNF) and Nerve Growth Factor (NGF) [87,88,89,90,91]. CREB-regulated BDNF is reduced in AD [88,91,92,93,94] and DS [95,96], and the magnitude of this reduction correlates with cognitive alterations [97]. Regarding NGF, the levels of this neurotrophin are reduced in DS and AD [98,99]. Dysfunction of NGF signalling induces the accumulation of APP C-terminal fragments and Aβ aggregation [100]. Aβ downregulates CREB-mediated transcription [101,102], resulting in synaptic loss and neurodegeneration [92,103,104]. Thus, BDNF/NGF and CREB downregulation could be one of the mechanisms implicated in the cognitive decline observed in AD [82,92,105,106,107].

## 3. Neurofibrillary Tangles

The accumulation of NFTs composed of hyperphosphorylated tau protein is one of the most characteristic neuropathological characteristics of AD in individuals with or without DS, and it also results from altered proteostasis [108,109,110]. Tau is an axonal phosphoprotein that promotes the self- assembly of tubulin into microtubules and its stabilization in neurons. Tau phosphorylation plays a physiological role in microtubule dynamics. However, hyperphosphorylation of this protein hampers its ability to bind to microtubules, leading to self-assembly and aggregation into NFTs [111,112]. This aberrant process impairs neurotransmission and increases cognitive decline. In fact, hyperphosphorylation of tau, even in the absence of Aβ aggregates, induces cognitive deficits [113].

Individuals with DS and murine DS models display aberrant tau phosphorylation earlier than normal subjects [114,115,116]. Alterations in different signalling pathways in AD individuals with or without DS are responsible for this pathological process. Similarly to what was previously described for amyloid plaque formation, kinases and phosphatases play a crucial role in tau hyperphosphorylation.

DYRK1A kinase can alter tau functioning by enhancing its phosphorylation and by altering tau splicing. DYRK1A phosphorylates tau at different residues [113,117,118], which alters microtubule assembly and stability in the brains of DS individuals and DS mouse models [115,119,120]. Also, DYRK1A phosphorylates NFAT [47] and provokes its inactivation [121,122]. The functional consequences of NFAT dysfunction and its relevance to AD neuropathology have been described in the previous section.

Moreover, alternative splicing of tau produces six different isoforms of this protein [123]. Two of them, 3R-tau (with three microtubule binding repeats) and 4R-tau (with four microtubule binding repeats) are generated by alternative splicing of tau at exon 10. In normal human brains, similar levels of both isoforms are expressed. However, in AD brains, 3R-tau is overexpressed and its levels correlate with aggravation of the disease. Also, the expression of this isoform is modulated by Aβ and by DYRK1A overexpression, which further enhances 3R-tau levels and increases the 3R-tau/4R-tau ratio [124,125].

Regarding another Hsa21-encoded kinase RCAN1, it has been demonstrated that increased levels of RCAN1.1 inhibit calcineurin activity. Calcineurin inhibition prevents the degradation of tau and enhances tau hyperphosphorylation [126,127]. As mentioned above, in DS, the calcineurin-NFAT signalling pathway is altered due to the overexpression of DYRK1A and RCAN1 [47], and a synergic effect exists between both kinases. DYRK1A phosphorylates RCAN1, increasing the ability of RCAN1 to inhibit calcineurin, leading to reduced NFAT transcriptional activity and enhanced tau phosphorylation [41].

In AD, the upregulation of the tau kinase GSK3β by RCAN1 can play a role in tau hyperphosphorylation and aggregation in NFTs [128,129,130]. Interestingly, both RCAN1.1 and GSK3β levels are elevated in the brains of AD patients, and these increases correlate with tau hyperphosphorylation [131] and Aβ production [132].

Other kinases and phosphatases not encoded by Hsa21 control tau phosphorylation. The brains of DS mouse models display increased levels of cyclin-dependent kinase 5 (CDK5) and decreased activity of the serine/threonine phosphatase 2A (PP2A) [133]. CDK5 is implicated in tau phosphorylation in AD [134,135] and DS brains [136]. Also, PP2A is involved in tau hyperphosphorylation in these conditions [137,138]. Thus, the downregulation of PP2A could be partially responsible for the abnormal tau phosphorylation in AD and DS [48,136,139,140,141,142].

mTOR signalling has also been demonstrated to be implicated in tau pathology in AD and DS. Individuals with DS show hyperactivation of mTOR signalling, which correlates with tau hyperphosphorylation, suggesting a role of this pathway’s dysregulation in tau neuropathology in AD and DS [56].

Sirtuin 1 (SIRT1) also interacts with mTOR and regulates mTOR phosphorylation. Reduced levels of SIRT1 are found in DS and AD [143] and mouse models of DS [144]. Furthermore, SIRT1 is a substrate of DYRK1A, which can promote tau accumulation by controlling its deacetylation process [144,145]. Thus, SIRT1 alteration might participate in the NFT deposition induced by aberrant mTOR signaling in DS.

Finally, basal forebrain cholinergic neurons present tau pathology in AD patients [146,147,148], since these neurons show an enhanced 3R-tau/4Rtau ratio [149]. Tau pathology in the basal forebrain cholinergic system occurs in the early stages of AD and is aggravated as the disease progresses [150,151,152], suggesting that tau pathology plays a role in cholinergic degeneration [153]. Acetylcholine receptors play an important role in aberrant tau phosphorylation in AD. While the activation of α7 nicotinic acetylcholine receptors (α7nAChR) facilitates tau phosphorylation, the activation of M1 muscarinic acetylcholine receptors (mAChR) prevents its phosphorylation [154,155,156,157,158]. In addition, nicotine induces tau phosphorylation in AD through the activation of nAChRs [157].

## 4. Cholinergic Neurodegeneration

One of the most relevant characteristics of AD neuropathology in individuals with and without DS is the degeneration of the basal forebrain cholinergic system [159,160]. The cholinergic system plays a critical role in different components of cognitive function such as attention, information processing, learning, and memory [159]. Altered cholinergic neurotransmission is one of the main determinants of dementia in AD [see 159] and the neuropathological sign that better correlates with the cognitive decline in this disorder [161,162].

AD brains lose increasing numbers of cholinergic neurons as the disease progresses [163,164]. Also, the main components of cholinergic signalling are affected in AD. The levels of the enzyme that catalyze the synthesis of acetylcholine (ACh) and choline acetyltransferase (ChAT) and of the enzymes that degrade ACh (i.e., acetylcholinesterase (AChE)), the vesicular acetylcholine transporter (VAChT) that transports ACh into the vesicles of mAChR and nAChR, are lower in AD and DS than in the normal population. ACh binding to these receptors is also decreased in both conditions [155,165,166,167,168,169].

Several mechanisms play a role in cholinergic neuron loss. Reduced expression of the neurotrophic factor NGF, its precursor proNGF, and their receptors TrkA and p75NTR are found in DS and AD [170]. These alterations can affect cholinergic neuron survival and ACh release [171].

A positive feedback mechanism between the degeneration of this population of neurons and other neuropathological characteristics of AD has been demonstrated. First, as mentioned above, this degeneration has a relevant role in tau pathology in AD. Second, Aβ peptides induce neurodegenerative changes at cholinergic terminals and can alter cholinergic activity, affecting NGF signalling and the consequent tau phosphorylation [172,173]. In turn, cholinergic neuropathology can aggravate Aβ pathology in AD [174].

## 5. Changes in Energy Consumption and Accumulation: Oxidative Stress, Mitochondrial Alterations, and Energy Metabolism

### 5.1. Oxidative Stress

Oxidative stress (OS) is one of the most important mechanisms implicated in the neuronal alterations found in DS and AD [175,176]. This process is involved in cellular redox homeostasis, synaptic plasticity, vesicle-mediated transport, neuroinflammation, protein folding and degradation, and signal transduction [177].

In DS, redox imbalance is caused by the enhanced production of reactive oxygen species (ROS) and the inhibition of antioxidant defense mechanisms [175,178]. The overexpression of different Hsa21 genes, which encode proteins that promote ROS production, plays an important role in the enhanced OS found in this syndrome [179,180,181]. One of these genes is *SOD1*, which encodes the enzyme that catalyzes the transformation of superoxide anions into molecular oxygen and hydrogen peroxide (H_2_O_2_). The increased activity of this enzyme in DS leads to the formation of high levels of H_2_O_2_ which are not adequately neutralized by the activity of the antioxidant enzymes catalase and glutathione peroxidase, which contributes to the redox imbalance [182].

The Hsa21 *RCAN1* gene also plays a role in OS in DS and AD, mainly through the regulation of mitochondrial function [183]. The brains of individuals with sporadic AD present an enhanced expression of RCAN1 [46]. OS induces the expression of RCAN1 via a calcineurin–NFAT-dependent mechanism [184], which inhibits calcineurin activity [185,186] and increases the stress response [183,187]. Also, Aβ enhances RCAN1 protein expression, reducing calcineurin through the induction of OS [46,186]. Altogether, these data suggest that a positive feedback mechanism exists between RCAN expression, OS, and Aβ pathology.

Another Hsa21 gene that plays an important role in oxidative stress is *APP*. As mentioned in previous sections, abnormal processing of the APP protein leads to enhanced levels of Aβ. These oligomers induce OS by increasing protein, lipid, DNA, and RNA oxidation [187,188], which leads to alterations in different biochemical and metabolic pathways implicated in AD neuropathology [188].

OS also contributes to alteration of the function of two neurotransmitter systems in AD, which are the targets of current pharmacological treatments of this disease. First, OS plays an important role in glutamate-mediated excitotoxicity in which excessive Ca^2+^ causes cell death [189]. AD is characterized by increased levels of HNE (a product of lipid peroxidation) bound to the glutamate transporter (GLT-1), which prevents the effective removal of glutamate from the synapse, thus promoting excitotoxicity [190]. Regarding the cholinergic system, the levels of HNE-bound ChAT are significantly increased by Aβ42 in AD [191]. Thus, OS can also contribute to alterations in ChAT activity in this disorder. 

Finally, ROS modifies the function of the mTOR pathway, which in turn can affect different components of OS. A feedback loop exists between OS and the mTOR pathway [192]. The role of OS in the altered function of the mTOR/autophagy axis in AD and DS has been demonstrated [193,194,195,196]. In the DS brain and DS mouse models, a link between protein oxidative damage and altered mTOR function has been demonstrated [194,195,196]. Because of the aforementioned role of mTOR in the regulation of proteostasis [197], the alteration of this system leads to Aβ and tau pathology in DS and AD [181]. Enhanced ROS, characteristic of these conditions, alters the regulation of autophagy. In turn, altered mTOR activity and reduced autophagy increase ROS production and oxidative damage in DS [195,196,198], thereby facilitating AD neuropathology.

### 5.2. Mitochondria

Mitochondria are highly metabolic organelles necessary for the maintenance of physiological redox signalling and neuronal activity [199]. Alterations in mitochondrial integrity increase ROS formation [200,201]. In turn, enhanced ROS levels also affect proper mitochondrial function [201]. Altered mitochondrial function plays a role in AD neuropathology including synapse and neuronal loss [177].

The oxidative phosphorylation (OXPHOS) system is the main energy provider to power the activity of mature neurons [202]. According to the “mitochondrial cascade hypothesis”, the origin of AD is a defect in the OXPHOS system [203]. Bioenergetics and Aβ are closely related. Aβ can reduce OXPHOS function and OXPHOS deficiency can increase Aβ production [204]. An OXPHOS defect has been reported in AD [14,205], which secondarily affects de novo pyrimidine biosynthesis and the plasma membrane remodeling of these patients [14]. This might explain the alterations in the composition and structure of neuronal membranes linked to the loss of synapses, which precedes neuronal loss in individuals with AD [206].

DS is also characterized by alterations in OXPHOS function. A reduction in the mRNA levels of several subunits of OXPHOS complexes has been found in DS brains [207,208,209,210,211]. This decline in mRNA levels was accompanied by a lower transcription of mtDNA-encoded genes [11,12]. The quantity of protein subunits for OXPHOS complexes is also reduced in DS brains [211,212,213,214,215]. A decrease in oxygen consumption [210,215,216,217,218,219] and a reduction in mitochondrial inner membrane potential are also characteristic of DS [12,216,218,219,220]. These alterations lead to a reduction in mitochondrial energy production and a lower mitochondrial function [12,215,217,218,219,220].

Some Hsa21 genes play a role in the OXPHOS function. For example, the overexpression of *DYRK1A* represses a transcriptional coactivator, peroxisome proliferator-activated receptor gamma coactivator 1 α (PGC-1α) that is a key modulator of mitochondrial biogenesis and OXPHOS function [221]. Also, in DS, overexpression of a transcriptional corepressor gene mapping to Hsa21, the nuclear receptor-interacting protein 1 (NRIP1), represses PGC-1α and decreases the mRNA levels of several OXPHOS-related genes [222].

The raptor–mTOR complex plays a role in mitochondrial activity and metabolism [223]. The activation of this complex stimulates the production of ATP by oxidative phosphorylation. In turn, the mTOR pathway is regulated by the redox status of the cell [224]. Thus, again, a positive feedback loop exists between mitochondrial redox status and mTOR activity [197]. As mentioned in previous sections, this pathway is dysregulated in AD and DS and the consequences of this dysregulation play different roles in neurodegeneration.

### 5.3. Energy Metabolism

One of the mechanisms proposed to be implicated in AD neurodegeneration is the impairment in energy metabolism [197,225]. The risk of developing AD is higher in individuals with obesity [177,226,227,228,229], type II diabetes [230,231,232,233], impaired glucose metabolism [227,233], and hyperlipidemia [234,235]. All of these conditions can cause impairments of brain cells and aggregation of Aβ [236,237]. Some authors have proposed that AD can be considered type III diabetes [238].

Brain insulin resistance is “the failure of brain cells to respond to insulin as they normally would, resulting in impairments in synaptic, metabolic, and immune response functions” [239]. Systemic insulin resistance is a crucial aspect of type II diabetes that contributes to inflammation and oxidative stress. However, brain insulin resistance can occur in the absence of systemic insulin resistance and type II diabetes. Also, so far, it has not been clarified whether systemic and brain insulin resistance affects cognition through the same mechanisms [239]. 

Alterations of the brain’s insulin resistance pathway have been associated with the development of AD [240]. AD and DS brains show reduced expression of insulin receptors (IR) and increased serine phosphorylation (inhibitory) of insulin receptor substrate 1 (IRS1) [240,241,242,243]. These changes produce alterations in neuronal survival and plasticity, protein synthesis and expression, cell differentiation, and synapse formation [244]. However, normal insulin levels can protect against Aβ toxicity and OS [240].

Also, AD patients display altered glucose transport due to the decreased levels of the glucose transporters GLUT2 and GLUT3 [245] and impaired glucose metabolism [246,247,248]. Indeed, decreased glucose catabolism is found in AD [249,250]. In addition to the production of ATP, the glucose metabolism provides energy and precursors for the biosynthesis of neurotransmitters such as GABA and glutamate and plays a role in autophagy [251,252]. Thus, alterations in glucose metabolism can affect neurotransmission and autophagy.

Different signalling pathways have been implicated in these alterations in energy metabolism in the brains of AD patients. First, the PI3-K/Akt/mTOR axis plays a role in the regulation of energy balance by modulating the response to insulin growth factors (IGFs) and epidermal-derived growth factors (EGFRs). Hyperactivity of the mTOR pathway produces insulin resistance [253] in the brains of AD individuals and mouse models of this disorder [67,73,253], playing a role in the aforementioned AD neuropathology. mTORC1 regulates protein synthesis, autophagy, mitochondrial function, lipogenesis, ketogenesis, and glucose homeostasis through the activation of IGF and EGFR [51]. Growth factors also activate mTORC1 through the Ras signaling pathway effectors ERK1/2 [254]. Also, mTORC2 activates Akt, while Akt modulates mTORC1 [197].

Another kinase implicated in the altered function of the mTOR pathway is AMPK, which regulates cellular metabolism in response to decreased intracellular ATP levels. AMPK and mTOR regulate autophagy [255]. While AMPK activates autophagy, mTOR reduces it. The induction of autophagy by AMPK reduces Aβ levels [66], while the activation of mTOR increases the levels of these peptides [256]. These data provide a further link between the mTOR pathway and altered metabolism in DS and AD.

## 6. Cellular Senescence

Cellular senescence, a homeostatic process which reduces proliferation and helps to prevent the propagation of damaged cells [257,258], is implicated in the neurodegenerative processes found in AD in individuals with or without DS [15].

Senescent cells are characterized by permanent arrest of the cell cycle [259,260], an increase in the synthesis and release of proinflammatory cytokines, (also called senescence-associated secretory phenotype (SASP)) [261], alterations in mitochondrial function, OS [262], changes in cellular metabolism [263], accumulation of DNA damage [264], changes in nuclear morphology and gene expression [265], and altered proteostasis [266]. As discussed in this review, all these changes can contribute to AD neurodegeneration.

Enhanced senescence has been found in AD and DS brains [15], and it has been proposed to play an important role in the onset and aggravation of AD neuropathology, including Aβ deposition [267], tau phosphorylation [268], increased release of proinflammatory cytokines [269,270] (see Section 7), and alterations in mitochondrial function and OS [176]. A positive feedback loop between cellular senescence and neurodegeneration has been proposed [15].

Additionally, in DS, the overexpression of some genes also induces cellular senescence. The triplication of the Ubiquitin-Specific Peptidase 16 (*USP16*) gene that encodes a histone H2-specific deubiquitinase plays a role in the enhanced senescence in DS [271]. In a mouse model of DS, the overexpression of this gene downregulates the Wingless and Int-1 (Wnt) signalling pathway, reducing stem cell renewal. USP16 activates Cdkn2a, which acts as a negative regulator of the Wnt signalling pathway. In turn, Wnt plays a crucial role in cellular senescence and aging in various tissues [272,273]. Besides, the USP16 enzyme regulates DNA damage repair by controlling the ubiquitination state of histone H2A. Overexpression of USP16 may induce excessive DNA damage accumulation, leading to acquisition of prematurely senescent phenotypes in different DS cell types [272,274].

## 7. Immune Response/Inflammation

Years before the appearance of Aβ plaques and NFTs, prominent neuroinflammation was present in the brains of individuals with AD and DS [7,275]. This enhanced neuroinflammation has been demonstrated to play a crucial role in the onset of neurodegeneration in these disorders. Neuroinflammation in DS and AD enhances the production of ROS and aggravates synaptic dysfunction, and Aβ and tau pathology [276,277], while amyloids aggregate NFTs and increase neuroinflammation [278,279].

The brains of AD and DS individuals and of mouse models of these conditions have higher levels of neuroinflammation due to microglia activation, which enhances the release of pro-inflammatory cytokines [7,270,275,280]. Among the inflammatory mediators that have been shown to have a role in neurodegeneration are interleukin-1 (IL-1), IL-6, and IL-17, which are upregulated in DS and AD [270,281,282]. Individuals with AD and mouse models of AD present increased activity of p38 Mitogen-Activated Protein Kinase (p38MAPK), a regulator of the release of cytokines [283]. p38MAPK increases the levels of a number of these cytokines in AD brains, including IL-6, IL-1, and Tumor necrosis factor-α (TNF-α) [281,282,284,285].

Among the mechanisms by which enhanced cytokine release aggravates neuroinflammation is their ability to enhance the expression of APP, the formation of Aβ oligomers, tau hyperphosphorylation, and ROS production [279,286]. However, as previously mentioned, neuroinflammation is not only a cause of neurodegeneration but also a consequence of it. In AD brains, Aβ and APP activate glial cells [287,288], which induces the release of proinflammatory mediators, including IL-1 and Interferon γ (IFNγ) [289]. Because of the high levels of these cytokines, the cells accumulate excessive levels of Aβ that are more likely to be aggregated [7]. Also, IL-1β can exacerbate Aβ expression by increasing BACE [290,291]. IL-1 also affects the activity of the Hypothalamic-Pituitary-Adrenal (HPA) axis, yielding an enhanced release of glucocorticoids [292]. Individuals with AD display hypercortisolism due to alterations in HPA regulation [293]. These high levels of glucocorticoids play a role in other alterations found in AD such as energy deficits [294], insulin resistance [295], and enhanced OS [296].

Cytokines are released by activated microglia, which can also regulate Aβ deposition by phagocytosis [297,298]. When Aβ induction is increased, these cells release inflammatory factors, which results in further activation of microglia and the enhanced release of cytokines and other neurotoxic factors [299,300]. In these circumstances, microglia migrate to Aβ and tau, surrounding them through special pathways and receptors such as CD14 and CD36 [301,302,303], further enhancing the production of pro-inflammatory factors, which damage healthy neurons.

One of the signalling pathways that is implicated in microglia activation is Wnt. This pathway is also implicated in tau hyperphosphorylation and synaptic loss [304]. Both the noncanonical (Wnt5a) and the canonical (Wnt3a) Wnts pathways are implicated in neuroinflammation in AD [304].

## 8. Changes in Cell Proliferation/Differentiation and Migration

AD is also characterized by reduced neurogenesis [9]. In DS brains, deficits in cell proliferation and differentiation into neurons are found from the early developmental stages and throughout the entire lifespan of the individual [305,306]. Because of the massive loss of different populations of neurons, the reduced regenerative capacity of the brains of individuals with AD with or without DS aggravates the progression of the disease.

Different signalling pathways are implicated in this deficient neurogenesis. As mentioned in Section 6, cellular senescence produces cell cycle arrest in AD and DS. In addition, several kinases and phosphatases encoded in Hsa21 play an important role in neurogenesis defects. First, the Hsa21 gene product DYRK1A plays a role in the altered cell proliferation, differentiation, and survival found in AD and DS through its interaction with different signalling pathways [305,306]. DYRK1A is a negative regulator of cell cycle progression because its overexpression promotes cell cycle exit [307]. Overexpression of DYRK1A also induces premature neuronal differentiation of neuronal progenitors, resulting in a depletion of mature neurons [307]. These altered proliferation and differentiation states induced by DYRK1A are due to its action on different signalling pathways.

One of the downstream DYRK1A pathways that has been implicated in cell cycle arrest is DREAM, a multisubunit complex that regulates quiescence [308]. DREAM complex formation occurs in the G0 phase after DYRK1A phosphorylation [309], which leads to an inhibition of cell proliferation. Also, the phosphorylation of cyclin D1 by DYRK1 inhibits neural cell proliferation and promotes premature differentiation by preventing entry into the S phase [310,311].

DYRK1A inhibits notch signalling, a pathway that controls neurogenesis by maintaining a pool of neuronal progenitor cells (NPCs) in the brain [312]. Thus, it might be implicated in the altered neurogenesis found after DYRK1A overexpression. Notch is overexpressed in the brains of AD and DS individuals [313,314]. The notch signalling pathway is also involved in promoting gliogenesis [315]. DS individuals exhibit an increased number of astrocytes and a reduced number of neurons when compared to the normal population, which provides support for the involvement of the notch signalling pathway in the neurogenic-to-gliogenic shift in DS brains.

Another DYRK1A target is NFAT, for which transcription is inhibited by DYRK1A [47]. Overexpression of DYRK1A and RCAN1 delays neurogenesis by their synergic action on the NFAT pathway [316].

One of the functions of the mTOR signalling pathway is the modulation of cell proliferation and survival [317]. Hyperactivation of this pathway can produce the apoptotic death of NPCs [318]. Thus, the aforementioned alterations in mTOR signaling in DS and AD brains might also be implicated in the neurogenesis defects found in these conditions.

Neurotrophins regulate neuronal survival, differentiation, and migration [319,320]. Among the downstream signalling pathways activated by neurotrophins are the MAPK, PI3K, and phospholipase C-γ (PLCγ) pathways [321,322]. BDNF, the most widely distributed neurotrophic growth factor in the CNS, is essential for the growth, differentiation, and survival of neurons [319,322]. Aβ decreases BDNF by lowering phosphorylated CREB protein. Reduced expression of BDNF is found in AD and DS brains, and it is thought to play a crucial role in the progression of this disease [92]. One of the mechanisms by which this reduced expression may operate is through the impairment of cell proliferation and differentiation.

In DS brains, the mitogenic Sonic Hedgehog (Shh) pathway plays a prominent role in neurogenesis impairment since alterations to this pathway reduce the proliferation of NPCs in different brain areas [323,324,325]. The APP gene plays an important role in cell cycle regulation [326] and is implicated in the altered Shh signalling found in DS [325]. The amyloid precursor protein intracellular domain (AICD) is a cleave product of APP. In DS, APP overexpression produces excessive levels of AICD, which upregulates transcription of the Shh receptor Ptch1 (Patched1). This receptor maintains the Shh pathway in a repressed state [324,325], impairing neurogenesis and aggravating neurodegeneration in DS. Thus, impairment of the Shh pathway due to APP-AICD-dependent Ptch1 overexpression may be a key mechanism that underlies the reduced proliferation and impaired maturation of neuronal precursors in DS and possibly in AD [327].

## 9. Alterations in Intercellular Signalling: Neurotransmitter Release, Synapses, and Receptors

### 9.1. Neurotransmitter Release

AD and DS are also characterized by alterations in cellular signalling due to multiple mechanisms. One of the most relevant implicates the DYRK1A and RCAN1 kinases, which have been demonstrated to impair neurotransmitter release. Overexpression of DYRK1A, as occurs in DS and AD, induces alterations in the serotoninergic, dopaminergic, and noradrenergic systems [328]. Because serotonergic transmission is related to GABA synthesis and the glutamatergic and monoaminergic systems interact [329], DYRK1A overexpression can participate in the widespread altered transmission seen in these conditions [330].

Additionally, one of the roles of RCAN1 is the control of neurotransmitter release [331]. Overexpression of this kinase reduces neurotransmitter secretion by impairing the outflow from vesicles [331]. These effects are likely to be due to the inhibitory activity that RCAN1 exerts over calcineurin activity [332], which regulates exocytosis and vesicle recycling [333]. Thus, it is likely that the altered expression of both DYRK1A and RCAN1 is implicated in the impaired intercellular signalling found in AD brains with or without DS.

### 9.2. Synapses

Years before the appearance of amyloid plaques and NFTs, a massive loss of synapses was evident in the brains of AD patients with or without DS. Alterations in multiple signalling pathways were implicated in this event. First, DYRK1A plays a critical role in synaptic dysfunction [334]. This kinase controls synaptogenesis through axon guidance [335] and the development and maintenance of neurites and dendritic spines [115,336], which are the first stages of synaptic formation. DYRK1A overexpression reduces neuronal dendritic growth and complexity [337] and inhibits the formation of dendritic spines [338].

DYRK1A also regulates synaptic vesicle formation. Overexpression of DYRK1A inhibits endocytosis [339] as well as the production of synaptic components implicated in synapse formation and maintenance [340] such as neuroligin 1 [341] and dynamin [342]. Finally, DYRK1A is also involved in synaptic transmission. Overexpression of this kinase impairs this process [338,343] partially through the modulation of CREB that is implicated in signal transduction pathways responsible for synaptic plasticity [307]. Thus, DYRK1A overexpression is also involved in the alterations of synapse formation, maintenance, and function found in these conditions.

RCAN1 also plays an important role in the synaptic dysfunction found in DS and AD brains. Similar to DYRK1A, RCAN1 mediates axon outgrowth by modulating the actin dynamics of the growth cone [344]. Overexpression of RCAN1 modifies the localization of synaptic proteins such as synaptophysin [345] and decreased phosphorylation of proteins necessary for synaptic plasticity such as CaMKII and ERK1/2 [346]. Calcineurin plays a crucial role in synaptic plasticity and endocytosis through the activation of its downstream targets NFATc, dynamin, and the Hsa21 encoded protein synaptojanin [347,348,349]. Thus, alterations in calcineurin because of RCAN overexpression in DS and AD are implicated in the synaptic dysfunctions found in these conditions.

Another Hsa21 gene that has been demonstrated to be implicated in synaptic alterations in DS and AD is the Down syndrome Cell Adhesion Molecule (*DSCAM*). This gene plays an important role in dendritic patterning, axon guidance and branching, and synaptic formation. Overexpression of DSCAM in mouse models and DS individuals inhibits dendritic branching [350,351] and synapse formation [352].

Intersectins (ITSNs) are a family of multi-domain adaptor proteins that regulate endocytosis, vesicle recycling, and cell signalling [353]. ITSNs regulate multiple signalling pathways including receptor tyrosine kinases (RTKs), GTPases, and phosphatidylinositol 3-kinase Class 2beta (PI3KC2β). The *ITSN1* gene is encoded in Hsa21 [354]. mRNA and protein levels are enhanced in DS and AD [355,356], and this gene is one of the most highly induced genes in AD brains [356]. Increasing evidence supports a role for this protein in the synaptic alterations found in these conditions. Both DS and AD are characterized by enlargement of the early endosomal compartment [357], a sign of altered endocytotic trafficking. This alteration leads to a reduced number of synaptic vesicles and their recycling, which resembles the effects of ITSN1 overexpression. Finally, ITSN1 is also involved in dendritic spine development through the regulation of different proteins [358].

The Wnt signalling pathway protects microglial synapse function and promotes the maturation of neuronal circuits [304]. However, under pathological conditions, such as AD, the canonical Wnt pathway is inhibited, leading to alterations in synapse number and function [359]. In AD, hyperphosphorylation of tau modifies synaptic function through modifications in the Wnt signalling pathway. Synapses damaged by Aβ are eliminated by microglia. However, at the same time, microglia release proinflammatory cytokines that can damage synapses [360,361] either directly or through activation of the Wnt receptor FZD [362]. In DS brains, these pro-inflammatory cytokines can alter the protein expression of synaptic markers (i.e., synapsin-1, PSD95, and GAD65/67) [363].

The AKT/mTOR pathway plays an important role in dendrite and spine morphogenesis, and synaptic transmission [57], partly through the release of cytokines [364], which in turn can activate the mTOR pathway. These events are implicated in the loss of synapses seen in DS and AD. Finally, the PI3K/AKT pathway, which can be induced by growth factors acting on their tyrosine kinase receptors, plays an important role in synaptic development [365,366].

In summary, the abnormal activation of multiple pathways and their synergic actions seem to be responsible for the early synaptic dysfunction found in DS and AD.

### 9.3. Receptors

Several lines of research demonstrate the important role of G-protein coupled receptors (GPCRs) in the altered signaling pathways found in AD and DS. In these conditions, GPCRs are implicated in tau hyperphosphorylation through several downstream kinases including GSK3β, CDK-5, and ERK signaling cascades [367]. An imbalance in tau phosphorylation mediated by GPCR-mediated kinases occurs in AD [368]. Several GPCRs have been associated with this imbalance including i) muscarinic ACh receptors [158], for which the number is reduced in AD, leading to enhanced phosphorylation of tau; ii) the CXCR2 and CC3 chemokine receptors, for which activation is implicated in the inflammatory response [367] and tau phosphorylation [369] and which are upregulated in AD [370]; and iii) the metabotropic glutamate receptor 2 (mGluR2) that activates the ERK pathway [371] and is overexpressed in AD leading to tau phosphorylation [367,372]. However, other receptors also play important roles in AD neuropathology [see 367].

In summary, in AD, in individuals with or without DS, alterations in intercellular signalling, including inhibited neurotransmitter release, a reduced number of synapses and alterations in their function, and altered expression of different GPCRs, interfere with neurotransmission, synaptic plasticity, and cognitive function and play important roles in the onset and aggravation of AD pathology.

## 10. Therapies Targeting Different Pathways Implicated in AD Pathology

Currently, there is no effective treatment to prevent or delay AD in individuals with or without DS, and the only approved drugs, AChE inhibitors and memantine, exert limited symptomatic benefits. Thus, a great effort is being made to search for strategies that prevent or delay the course of the disease. Because of the complex pathology that appears sequentially or simultaneously in AD, a great number and diverse types of therapeutic strategies that target the different alterations found in this disorder are being tested. One of the main problems encountered has been the inability to replicate in humans the efficacy of the different strategies demonstrated in preclinical studies [373]. This section summarizes the current state of the most relevant therapies that target different pathways mentioned in this review.

First, to prevent the formation of amyloid plaques, active and passive immunotherapies that avoid the formation of Aβ by inhibiting BACE that reduce the aggregation of these oligomers into plaques or that facilitate the clearance of Aβ peptides have been developed [374]. Although many of the clinical trials failed due to severe side effects or to its inefficacy, numerous new immunotherapies are currently being tested [374].

Also, several inhibitors of the DYRK1A kinase have been demonstrated to reduce the neuropathology and to improve the cognitive abilities of mouse models of DS and AD [334]. One of them, (-)-Epigallocatechin gallate (EGCG), has been approved for use in the young-adult DS population. However, its ability to enhance the cognitive abilities of these individuals is very controversial, and so far, this molecule has not been tested in individuals with DS and AD. Besides, concerns have been raised about the safety of chronically inhibiting DYRK1A because of its multiple roles in numerous signalling pathways [334].

Although accumulated evidence indicates that RCAN1 might be a potential target for the treatment of AD and DS, so far, a drug able to inhibit RCAN1 has not been developed. However, Zmijewski et al. [375] demonstrated that fish oil supplementation reduced the levels of this protein in mice. Nonetheless, an important issue to take into account with compounds that inhibit calcineurin is that they are immunosuppressive. The therapeutic use for organ transplantation of calcineurin/NFAT inhibitors is associated with severe side effects [376].

Another potential therapeutic target for AD is the mTOR pathway. Numerous studies have demonstrated the ability of mTOR inhibitors, including rapamycin and its analogs, to reduce Aβ load, tau pathology, and cognitive decline in mouse models of AD. However, because these inhibitors induce adverse effects due to the role of mTOR in cell growth and proliferation, metabolism, and protein synthesis, they have not been tested in humans [377].

As explained in previous sections, CREB activation is reduced in AD, resulting in a synaptic and memory impairment. Thus, different strategies to enhance CREB activity have been tested in AD models. Among them, the phosphodiesterase 4 inhibitor rolipram [378] and dietary supplementation with different procyanidins, the main group of flavonoids, have been demonstrated to rescue different neuropathological characteristics of AD and to enhance cognition in animal models of this disorder [379]. However, because of the great number of roles that CREB plays in many tissues, chronic CREB activation could induce important adverse effects [378].

Among the most promising strategies to treat AD are the ones that target tau pathology. Different drugs have been developed to reduce tau translation, posttranslational modifications, aggregations, and impairments in clearance (see [380]). Besides the toxic effects that tau exerts on cells, it is also a mediator of Aβ toxicity; thus, reducing tau pathology could also help to minimize the main hallmark of AD. Despite the efficacy of some strategies that reduce tau expression such as small interfering mRNAs (siRNA) in preclinical models, no clinical trials have been performed in the AD population. Among the drugs that target tau protein modifications are (i) phosphatase inhibitors, such as the NMDA receptor antagonist memantine that produces benefits in AD patients and sodium selenate that increases PP2A activity and is currently been evaluated in a phase II clinical trial; (ii) kinase inhibitors, such as the CDK5 inhibitors flavopiridol and roscovitine that has not been tested in clinical trials in AD patients, tideglusib that does not produce improvements in the AD population and lithium chloride, and a GSK3β inhibitor, which stabilized the cognitive symptoms in AD patients; (iii) drugs that inhibit tau acetylation such a salsalate, a small-molecule NSAIDs; (iv) drugs that inhibit tau deglycosylation such as MK-8719; and (v) molecules that inhibit tau truncation. However, the efficacy of the last three strategies has not been demonstrated in clinical trials. Inhibitors of tau aggregation such as methylene blue and curcumin did not produce any clinical benefits in the AD population [380].

Finally, different tau active and passive immunotherapies have been developed. Similar to what was describe in the case of amyloid immunotherapies, although the results of preclinical studies were very promising, important side effects and low efficacy prevented its use in the AD population. However, several clinical trials trying to overcome these issues are being performed [380].

Growth factors such as NGF and BDNF have been proposed as an aid to prevent cholinergic neurodegeneration and other symptoms of AD [381]. Intranasal administration of NGF reduced cholinergic loss and improved cognition in animal models of AD and a clinical trial are currently being performed to assess its efficacy in humans with this condition. Regarding BDNF, preclinical studies in mouse models of DS have also proven to reduce cholinergic loss as well as other AD-related alterations. Interestingly, some of the approved drugs for the symptomatic treatment of AD such as memantine and donepezil increase BDNF levels [381].

Regarding oxidative stress, similar to what has been described with other therapeutic strategies, different preclinical studies have demonstrated the efficacy of numerous antioxidants (e.g., melatonin, Vitamin E, folinic acid, and different mixtures of vitamins and minerals) and clinical trials performed in individuals with AD with or without DS failed to find any benefit on the cognitive status of these patients or in their neuropathological status [186,382]. It is possible that, in the case of DS individuals, because oxidative stress is present from early developmental stages, the administration of antioxidants in later life stages is not able to rescue other well-established neuropathological signs such as amyloid plaques, NFTs, or synapse loss.

Several compounds that target the mitochondrial alterations found in AD such as Mito Q, Skulachev (SkQ1), melatonin, and Sezto–Shiller (SS) tetrapeptide SS31 reduce the neurodegenerative characteristics of mouse models of this disorder and are good candidates to be tested in clinical trials [383].

Antidiabetic drugs have been demonstrated to rescue most of the alterations found in mouse models of AD [384]. Both hypoglycemic agents (including insulin, sulphonylureas, and glinides) and antihyperglycemic agents (including metformin, thiazolidinediones, dipeptidyl peptidase (DPP) IV inhibitors, Glucagon-like peptide-1 (GLP-1) analogs, GLP-1 receptor agonists, and Sodium-Glucose co-transporters (SGLT)-2 inhibitors) reduce protein aggregation, neuroinflammation, and oxidative stress and enhanced neurogenesis, synaptic plasticity, and cognition in AD rodents [384]. Furthermore, clinical trials have demonstrated that intranasal insulin, sulphonylureas metformin, and the GLP-1 analog liraglutide enhance cognition in AD patients [384]. Thus, antidiabetes drugs are currently one of the most promising strategies to treat AD.

The reduction of neuroinflammation in AD patients has been proposed to be a promising stragegy to treat this disease. Chronic nonsteroidal anti-inflammatory drug (NSAID) consumption has been consistently associated with reduced risk for AD [385], and chronic ibuprofen or naproxen consumption delays the progression from mild cognitive impairment to AD. However, short-term treatments with NSAIDs do not reduce the risk of developing AD. Although buprofen treatment reduces amyloid accumulation and tau in these patients, NSAIDs might only be effective in ApoE4 carriers [385].

Another strategy to reduce neuroinflammation in AD brains is to convert microglia from an inflammatory to a phagocytic phenotype that can enhance the clearance of Aβ. One of the drugs that exerts this effect in mouse models of AD is jujuboside A [386]. However, more evidence of its effects must be obtained in preclinical studies before performing clinical trials.

Finally, it has been proposed that a combination of therapeutic approaches targeting different pathological aspects of the disease would be more effective. Various clinical trials combining different disease-modifying therapies and symptomatic therapies are being performed in individuals with AD [387]. This strategy has been extremely useful in the treatment of other complex diseases such as HIV.

## 11. Concluding Remarks

**Figure 1:**
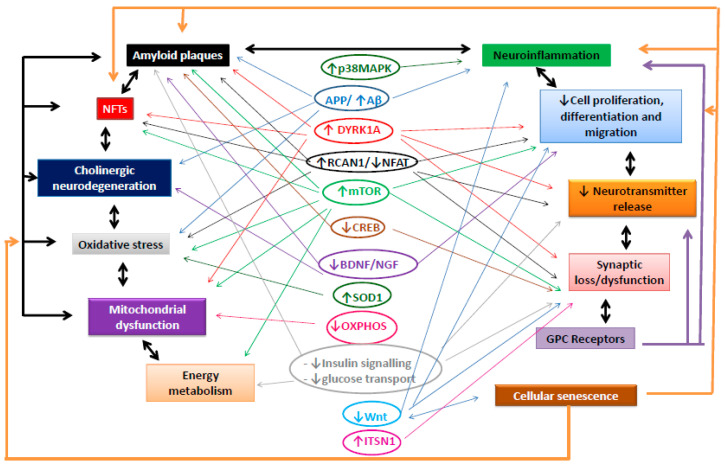
Graphical display of the main pathways (circled) implicated in each pathological characteristic of AD (squared) as well as their interconnections. Line and arrow colors depict the influence of the different signalling pathways and/or pathological characteristics circled or squared using the same color on other pathways or systems. Black arrows represent the feedback loops between the main pathological characteristics of AD. ↑: up-regulated, ↓down-regulated.

Two of the main neuropathological characteristics of AD are the accumulation of amyloid plaques and NFTs. However, numerous mechanisms that appear years earlier than these alterations play a crucial role in the onset and aggravation of this disease. These earlier events include neuronal and synaptic loss and dysfunction, enhanced OS, mitochondrial dysfunction, altered energy metabolism, cellular senescence and neuroinflammation, reduced neurogenesis, and altered neurotransmission. For these reasons, AD has been proposed to be a disease with a complex etiology in which these earlier alterations participate in the appearance and accumulation of plaques and tangles, which in turn aggravate the earlier pathological events in a positive feedforward loop. Also, as described in this review, these pathological mechanisms are interrelated. Numerous signalling pathways that regulate these events are altered in AD. Interestingly, many of these pathways are implicated in multiple AD-related neuropathologies. Besides, in many cases, a synergic effect and/or an interaction between these pathways exist. Among the most relevant examples are the numerous adverse effects found in AD brains due to the overexpression of the Hsa21-encoded DYRK1A and RCAN1 kinases or the mTOR pathway and their interactions. Table 1 summarizes the main signalling pathways implicated in each of the neuropathological characteristics of AD mentioned in this review, and Figure 1 depicts these pathways as well as their interconnections. The complex scenario of AD etiopathology suggests that the development of therapies designed to treat this disorder should target the molecular pathways implicated in multiple altered events of the disease. Finally, because of the high prevalence and early appearance of AD in the DS population and the multiple common mechanisms found in both conditions, DS can be considered a useful model to study AD etiopathology and to search for new therapeutic strategies.

## 12. Key Summary Points

- AD, in individuals with or without DS, is a disease with a complex set of neuropathological signs.
- Numerous signalling pathways are implicated in the onset and aggravation of this neuropathology.
- The same signalling pathway often plays a role in the appearance or progression of different signs of AD.
- In many cases, synergic effects and feedback loops exist between these pathways.
- Because of the complex etiopathology of AD and the interrelation between the factors responsible for the symptoms of the disease, therapeutic approaches should combine different targets.

## Figures and Tables

**Table 1:** Signalling pathways implicated in the main neuropathological characteristics of Alzheimer’s disease in individuals with and without Down syndrome.

Neuropathological Characteristic	Signalling Pathway	Up- or Downregulation	Pathophysiological Role in AD
**Amyloid plaques**	APP [31]	↑ in DS and AD	Generation of Aβ oligomers
	DYR1A [37,38,39,40,47]	↑ in DS and AD	Aβ degradation, APP phosphorylation
	RCAN1/NFAT [42,43,45,46,47,48]	↑ RCAN/↓ NFAT in DS and AD	Mediation of Aβ-induced neuronal death, disruption of Ca^2+^ homeostasis
	PIK3/Akm/mTOR [51,52,55,56,57,64,65,66,67,68,74,75]	↑ in DS and AD	Contribution to Aβ generation and aggregation, inhibition of autophagy, reduction of Aβ clearance
	CREB [80,81,82,83,84,85,86]	↓ in DS and AD	Induction of synaptic loss by Aβ
	BDNF/NGF [88,92,93,98,99,100]	↓ in DS and AD	Accumulation of APP C-terminal fragments and aggregation of Aβ
**Neurofibrillary tangles**	DYRK1A [117,118,119,120,121,122,123,124,125]	↑ in DS and AD	Modifications in tau splicing and enhancement of tau phosphorylation
	RCAN1/NFAT [126,127,128,129,130,131]	↑ RCAN/↓ NFAT in DS and AD	Prevention of tau degradation and enhancement of tau phosphorylation
	CDK5 [133,134,135,136]	↑ in DS	Enhancement of tau phosphorylation
	PP2A [137,138,139,140,141]	↓ in DS	Enhancement of tau phosphorylation
	mTOR/SIRT1 [56,143,144,145]	↑ mTOR/↓ SIRT in DS and AD	Enhancement of tau phosphorylation, promotion of tau accumulation
	Cholinergic system [156,157,158,159,160,161,162,163,164,165,166,167,168,169]	↓ in DS and AD	Tau pathology in cholinergic neurons that aggravates neurodegeneration
**Cholinergic neurodegeneration**	NGF/proNGF/TrkA/p75NTR [170,171,172,173]	↓ in DS and AD	Reduction in survival of cholinergic neurons
	Aβ [172,173,174]	↑ in DS and AD	Facilitation of cholinergic neurodegeneration
**Oxidative stress**	SOD1 [182]	↑ in DS	Induction of Redox imbalance
	RCAN1/NFAT [46,183,184,185,186,187]	↑ RCAN/↓ NFAT in DS and AD	Alterations in mitochondrial function and increase in ROS production
	APP/Aβ [188,191]	↑ in DS and AD	Enhancement of lipid, DNA, and RNA oxidation
	Glutamatergic system [189,190]	↑ in AD	Promotion of OS-induced excitotoxicity
	Cholinergic system [191]	↓ in DS and AD	Aβ-induced enhancement of OS in cholinergic neurons
	mTOR [192,193,194,196,198]	↑ in DS and AD	OS disruption of mTOR function and mTOR enhancement of oxidative damage
**Mitochondrial dysfunction**	Enhanced oxidative stress [191,200,201]	↑ in DS and AD	Enhancement of ROS-mediated disruption of mitochondrial integrity and function
	OXPHOS [203,204,205,206,209,210,211,212,213,214,215,216,217,218,219,220,221,222]	↓ in DS and AD	Enhancement of Aβ production, alterations in cell membranes and synapses, reduction in mitochondrial inner membrane potential, reduction in energy production, and lower mitochondrial function
	Raptor/mTOR [197,223,224]	↑ in DS and AD	Alterations in mitochondrial activity and metabolism
**Energy metabolism**	Insulin signaling [241,242,243]	↓ in DS and AD	Alterations in energy metabolism, impairment of neuronal activity, plasticity and survival, and facilitation of Aβ aggregation
	Glucose transport and metabolism [245,246,247,248,249,250]	↓ in DS and AD	Reduction in energy for synaptic transmission and neurotransmitter biosynthesis, alterations in autophagy
	PI3-K/Akt/mTOR [65,67,253–256	↑ in DS and AD	Dysregulation of energy balance, induction of insulin resistance, altered autophagy
**Cellular senescence**	Release of proinflammatory cytokines [269,270]	↑ in DS and AD	Induction of cellular senescence and enhancement by senescence
	Oxidative stress and mitochondrial dysfunction [15,262]	↑ in DS and AD	Induction of cellular senescence and enhancement by senescence
	Proteostasis (Aβ and tau) [267,268]	↑ in DS and AD	Induction of cellular senescence and enhancement by senescence, induction of cellular senescence and enhancement by senescence
	USP16-Wnt [271,272,273]	↑ UPS16 in DS/ ↓ Wnt in DS and AD	Induction of senescence through DNA damage, downregulation of the Wnt pathway reducing stem cell renewal
**Immune response/inflammation**	p38MAPK [281,282,283,284,285]	↑ in DS and AD	Increase in release of cytokines
	Aβ/APP [287,290]	↑ in DS and AD	Increase in release of cytokines which further aggravates Aβ pathology
	HPA [292,293]	↑ in AD	Cytokines produce excessive activation of the HPA, which aggravates the energy deficits and enhances OS
	Wnt [304]	↓ in DS and AD	Altered microglia activation, enhancement of neuroinflammation, tau hyperphosphorylation, and synaptic loss
**Cell proliferation/differentiation and migration**	DYRK1A [305,306,307]	↑ in DS and AD	Induction of cell cycle exit, premature differentiation or precursors resulting in a reduced number of adult neurons
	DYRK1A/DREAM [308,309]	-	Inhibition of cell proliferation due to cell cycle arrest
	DYRK1A/Cyclin D1 [310,311]		Inhibition of proliferation and promotion of premature differentiation, prevention the entry into the S phase of the cycle
	DYRK1A/Notch [312,313,314]		Inhibition of notch signaling that controls neurogenesis, induction of a shift from neurogenic to glionenic fate of progenitors
	DYRK1A/NFAT [316]		Delay of neurogenesis by the synergic effect with RCAN1
	mTOR [317,318]	↑ in DS and AD	Apoptotic death of NPCs
	BDNF [92,322]	↓ in DS and AD	Impairment of cell proliferation and differentiation
	Shh [323,324,325]	↓ in DS	Impairment of proliferation of NPCs
	APP [327]	↑ in DS and AD	Alterations in cell cycle regulation, neural precursor maturation
**Neurotransmitter release**	DYRK1A [328,330]	↑ in DS and AD	Reductions in neurotransmitter synthesis and release
	RCAN1 [331,332,333]	↑ in DS and AD	Reductions in neurotransmitter synthesis and release
**Synapses**	DYRK1A [334,335,336,337,338,339,340,341,342,343]	↑ in DS and AD	Impairment in dendritic growth and complexity; dendritic spine formation; reduction of synaptic components necessary for synapse formation, maintenance, and functioning
	RCAN1/NFAT [344,345,346,349]	↑ RCAN/↓ NFAT in DS and AD	Modification of the localization of synaptic proteins, decreased phosphorylation of proteins necessary for synaptic plasticity
	DSCAM [351]	↑ in DS and AD	Inhibition of dendritic branching and synapse formation
	ITSN [353,355,356,357,358]	↑ in DS and AD	Enlargement of the early endosomal compartment, altered endocytic trafficking, leading to a reduced number and recycling of synaptic vesicles
	Wnt [359,362]	↓ in DS and AD	Alterations in synapse number and function
	PI3K/AKT/mTOR [365,366]	↑ in DS and AD	Loss of synapses partly mediated by enhanced cytokine release, impairment of synaptic development
**Receptors**	Muscarinic ACh receptors [158]	↓ in DS and AD	Impairment of cholinergic transmission: the loss of these receptors is mediated by tau phosphorylation
	CXR2 and CC3 chemokine receptors [369,370]	↑ in AD	Enhancement of tau phosphorylation ad cytokine release
	mGluR2 receptors [371]	↑ in AD	Enhancement of tau phosphorylation
	Other GPCRs [367]	-	Alteration of neurotransmission by different mechanisms including tau phosphorylation, increased cytokine release, and aggravation of amyloid pathology

Aβ: β-amyloid; ACh: acetylcholine; AD: Alzheimer’s disease; APP: Amyloid Precursor Protein; BDNF: Brain-Derived Neurotrophic Factor; CREB: cAMP Response Element-Binding protein; CDK5: Cyclin-Dependent Kinase 5; DS: Down syndrome: DSCAM: Down syndrome Cell Adhesion Molecule; DYRK1A: Dual Specificity Tyrosine-Regulated Protein Kinase 1; GPCR: G-protein coupled receptors; HPA: Hypothalamic-Pituitary-Adrenal axis; ITSN: Intersectin; mGluR2: metabotropic glutamate receptor 2; mTOR: Mammalian Target of Rapamycin; NFAT: Nuclear Factor of Activated T cells; NGF: Nerve Growth Factor, OXPHOS: Oxidative Phosphorylation; p38MAPK: p38 Mitogen-Activated Protein Kinase; PIK3: Phosphoinositide 3 Kinase; PP2A: Phosphatase 2A; RCAN1: Regulator of Calcineurin 1; Shh: Sonic Hedgehog; SIRT1: Sirtuin 1; SOD1: Superoxide Dismutase; USP16: Ubiquitin-Specific Peptidase 16; Wnt: Wingless and Int-1; ↑: up-regulated, ↓down-regulated.
